# Evaluating the Expression of Candidate Homeobox Genes and Their Role in Local-Site Inflammation in Mucosal Tissue Obtained from Children with Non-Syndromic Cleft Lip and Palate

**DOI:** 10.3390/jpm11111135

**Published:** 2021-11-02

**Authors:** Nityanand Jain, Mara Pilmane

**Affiliations:** Department of Morphology, Institute of Anatomy and Anthropology, Rīga Stradiņš University, LV-1007 Riga, Latvia; mara.pilmane@rsu.lv

**Keywords:** cleft lip and palate, inflammation, immunohistochemistry, in-situ hybridization, *HOXB3*, *DLX4*, *MSX2*, *PTX3*

## Abstract

Craniofacial development including palatogenesis is a complex process which requires an orchestrated and spatiotemporal expression of various genes and factors for proper embryogenesis and organogenesis. One such group of genes essential for craniofacial development is the homeobox genes, transcriptional factors that are commonly associated with congenital abnormalities. Amongst these genes, *DLX4, HOXB3,* and *MSX2* have been recently shown to be involved in the etiology of non-syndromic cleft lip and palate. Hence, we investigated the gene and protein expression of these genes in normal and cleft affected mucosal tissue obtained from 22 children, along with analyzing their role in promoting local-site inflammation using *NF-κB*. Additionally, we investigated the role of *PTX3,* which plays a critical role in tissue remodeling and wound repair. We found a residual gene and protein expression of *DLX4* in cleft mucosa, although no differences in gene expression levels of *HOXB3* and *MSX2* were noted. However, a significant increase in protein expression for these genes was noted in the cleft mucosa (*p* < 0.05), indicating increased cellular proliferation. This was coupled with a significant increase in *NF-κB* protein expression in cleft mucosa (*p* < 0.05), highlighting the role of these genes in promotion of pro-inflammatory environment. Finally, no differences in gene expression of *PTX3* were noted.

## 1. Introduction

The processes of embryogenesis, organogenesis, and subsequent cell differentiation are highly regulated and tightly controlled by multiple genes which are expressed in an orchestrated manner in different tissues. Homeobox genes are one such group which are deeply involved in embryonic patterning and cell differentiation. These genes possess a specific DNA sequence called the “homeobox” which encodes for proteins called the “homeodomains” [1,2,3]. Homeodomains, when expressed, recognize, and bind to specific DNA sequences which allows them to regulate the expression of other genes and control the process of organogenesis [4]. Due to their role and functionality as a transcription factor, several homeobox genes have been associated with congenital abnormalities [5]. About 230 homeobox genes have been discovered in the human genome [6,7] which are classified into gene families (containing similar genes), which are further clustered into 11 gene classes (containing similar families) [3]. The largest and most relevant gene class for craniofacial development is perhaps the ANTP (Antennapedia) class, comprising of 37 gene families divided into two subclasses based on the chromosomal location of the genes—HOXL and NKL. The HOXL (HOX-linked) subclass consists of 14 gene classes which are concentrated in two fourfold paralogous regions while the NKL (NK-linked) subclass consists of remaining 23 gene classes which are rather more dispersed [3].

Within the NKL subclass, two gene families—DLX (Distal-Less homeobox) and MSX (Muscle segment homeobox homolog/Msh homeobox) have been implicated in non-syndromic cleft lip and palate. Amongst the DLX family, the *DLX4* gene is one of the latest genes which have been shown to play a role in clefting. The gene has been shown in murine models to be expressed in the mesenchyme derived from neural crest cells in the mandibular arch, the lower jaw primordia [8]. In human tissues, the gene expression quantification and identification has proven to be difficult due to non-expression in normal adult tissues [9]. However, the gene has been found to be expressed in different cancer cells including cells from breast [10], lung [11], prostatic [12] and colorectal cancer [13]. Additionally, in two recent studies, the gene has been associated with both non-syndromic [14] and syndromic forms of cleft lip and palate [15].

In the MSX family, mutations in the *MSX2* gene have been shown to be associated with clefting in the Brazilian population [16]. Furthermore, in a sequencing study, point mutations in the *MSX2* gene were found to be common amongst patients with bilateral cleft lip and palate and with a positive family history [17]. The gene expression is usually detectable by the middle of the seventh embryonic week in the mandibular and maxillary bone primordia, Meckel’s cartilage, and primordial tooth germs [18,19]. Moreover, the gene is essential for skeletal bone growth and differentiation since the overexpression of the gene impedes osteoblast differentiation while antisense inhibition promotes differentiation [19,20,21]. The *HOXB3* (homeobox B3) gene, another candidate gene for non-syndromic cleft lip and palate, is a member of the HOX3 family (part of the HOXL subfamily) that has been shown to transcriptionally regulate the expression of *JAG1* gene (Jagged Canonical Notch Ligand 1) in a synergistic manner during pharyngeal arch development [22]. Furthermore, the co-expression of *HOXB3* and *JAG1* has been demonstrated to affect the migration of the neural crest cells into the pharyngeal arches, which leads to neural crest deficiency and craniofacial defects [22].

Mutations and disruptions in the spatiotemporal expression of the above genes leads to incomplete development of the orofacial structures which in turn disrupts the functioning of the immune system and reparative processes [23]. Such disruption promotes local site inflammation due to an imbalance of the cytokines (pro vs. anti) which hinders normal tissue remodeling during wound healing [24]. Additionally, proinflammatory cytokines like IL17 and IL6, regulatory cytokine IFN-γ (interferon gamma) and anti-inflammatory cytokine IL4 modulate immunosteogenesis by actively participating in the process of osteogenesis [23]. Children with cleft lip and palate show disturbances in the immunosteogenesis during embryogenesis which affects the characteristics and the course of the disease postnatally [25]. These disturbances also create certain difficulties in the rehabilitation of such patients, which makes it essential to investigate the role of the gene-gene interactions, especially between causative genes and cytokine regulators. In one of our previous studies with the same cohort as in the current study, we showed the precise imbalance in the cytokine expression in the cleft mucosal tissue [26]. We reported an increase and constant expression of pro-inflammatory cytokines like IL-2,6,13 and TNF-α (tumor necrosis factor) which are known to stimulate osteogenesis. This was coupled with a decrease in anti-inflammatory cytokines like IL-4 and IL-10, which are known to downregulate osteogenesis. Interestingly, a strong correlation between IL-6 and IFN-γ gamma expression was noted, which indicated preferential Th1 pathway activation [26]. However, in the previous study we didn’t investigate the role of the master regulator of cytokine expression—*NF-κB* (Nuclear Factor Kappa Beta).

*NF-κB* is a family of five inducible transcription factors (p50/p105, p52/p100, p65 or RelA, c-Rel, and RelB) which regulates the immune and inflammatory responses [27,28]. These transcriptional factors are normally sequestered in the cytoplasm by a family of inhibitory proteins called IκB family members [29] and can be activated by two pathways—classical or canonical pathway and alternative or non-canonical pathway. In the classical pathway, when cells are stimulated by agents like inflammatory cytokines (TNF-α and IL-1), growth factors, mitogens, microbial components, and stress, IκB undergo phosphorylation which frees NF-κB (predominantly p50/RelA and p50/c-Rel dimers) to translocate into the nucleus and bind to specific DNA sequences [30,31]. In the non-classical pathway, very specific stimulants (ligands of a subset of tumor necrosis factor receptor superfamily members) stimulates p100, the NF-κB2 precursor protein [29,32]. The stimulants lead to phosphorylation and ubiquitination of p100, which then leads to the formation of a heterodimer of RelB/p52 which also translocate into the nucleus.

Finally, the soluble pattern recognition receptor *PTX3* (long-pentraxin 3) has been gaining traction and attention for the role it plays in innate immunity, tissue remodeling, and tumorigenesis. Like *NF-κB*, *PTX3* expression is also inducible by the same pro-inflammatory cytokines (whilst being downregulated by IL-4) in a variety of different cell types, including fibroblasts and endothelial cells, myeloid cells such as monocytes, macrophages, and dendritic cells (DCs), adipocytes, epithelial cells etc. [33,34,35]. In fact, epigenetic studies have demonstrated that NF-κB (especially RelA) binds specifically to an upstream enhancer (called Enhancer 2) of the *PTX3* gene after TNF-α stimulation in macrophages, highlighting the regulatory connection between the two [36]. Apart from mediating inflammatory responses, *PTX3* is heavily involved in the *FGF/FGFR* (fibroblast growth factor/FGF receptor) pathways [37]. It is known that *PTX3* possess FGF2-binding and inhibitory capacity (basic fibroblast growth factor/bFGF) which confers it anti-angiogenic properties [38,39]. In our last study (again, with the same cohort as the present study) we showed that *FGF2* was over-expressed in cleft mucosal tissue and hypothesized on the possible interaction and role of *PTX3* in cleft tissue [40].

Hence, in the present study, we investigated the gene (using CISH—chromogenic in-situ hybridization) and protein (using IHC—immunohistochemistry) expression of three recently implicated homeobox genes (*DLX4, MSX2*, and *HOXB3*) in normal and cleft affected mucosal tissue. Further, we investigated the interactions of these genes with *NF-κB* to understand the underlying mechanisms of local-site inflammation. Additionally, we aimed to understand the role and interactions of *PTX3* in cleft-affected mucosal tissue, providing us with better insights in the pathogenesis and manifestations of cleft lip and palate.

## 2. Results

### 2.1. Gene and Protein Expression Analysis of DLX4 Gene in Control and Cleft Tissue

**Figure 1:**
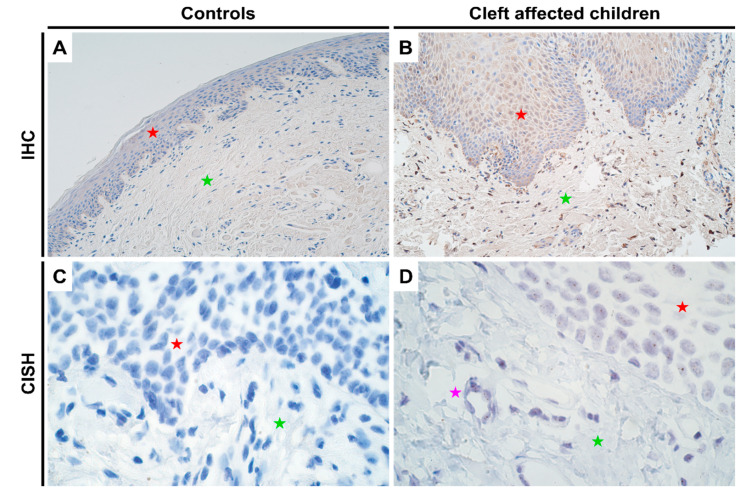
Expression of *DLX4* gene visualized using IHC and CISH methods in controls and cleft patients. Protein expression of the *DLX4* gene was visualized using immunohistochemistry (IHC) in (**A**) controls and (**B**) cleft affected children. (**A**) No positively stained (0) structures are visible in tissue obtained from controls; (**B**) Moderate number (++) of weakly stained epitheliocytes and distinctly stained connective tissue cells are seen in the cleft tissue obtained from a five-month-old girl. Original magnification, 200×. Gene amplification levels of *DLX4* gene was visualized using chromogenic in-situ hybridization (CISH) in (**C**) controls and (**D**) cleft affected children. (**C**) No gene expression and amplification (0) are seen in the tissue obtained from controls; (**D**) No gene amplification (0) is seen; however, gene expression is seen by brown dots in the nucleus of the epithelium and endothelium cells in the cleft tissue obtained from an eight-month-old boy. Original magnification, 1000×. Red star indicates epithelium; green star indicates connective tissue, while purple star indicates endothelial cells.

Since *DLX4* is not expressed in normal adult tissues [9], the CISH assay also showed no positive results in the control tissues (Figure 1C). However, in the cleft mucosal tissue, gene expression was detectable by CISH, though no gene amplification was seen (Figure 1D). In terms of protein expression, as expected controls again showed no positive IHC results (Figure 1A; Supplementary Table S1) whilst the cleft affected mucosal samples showed a significant increase in protein expression in both epithelium (*p* = 0.000) and connective tissue cells (*p* = 0.001) with a mean IHC semi-quantitative grade of 1.7+ and 1.6+, respectively (Figure 1B and Table 1; one-sample Wilcoxon signed rank test). No significant differences in terms of protein expression were observed between epithelium and connective tissue cells in cleft tissue (*p* = 0.565; paired Wilcoxon signed rank test).

### 2.2. Gene and Protein Expression Analysis of HOXB3 Gene in Control and Cleft Tissue

**Figure 2:**
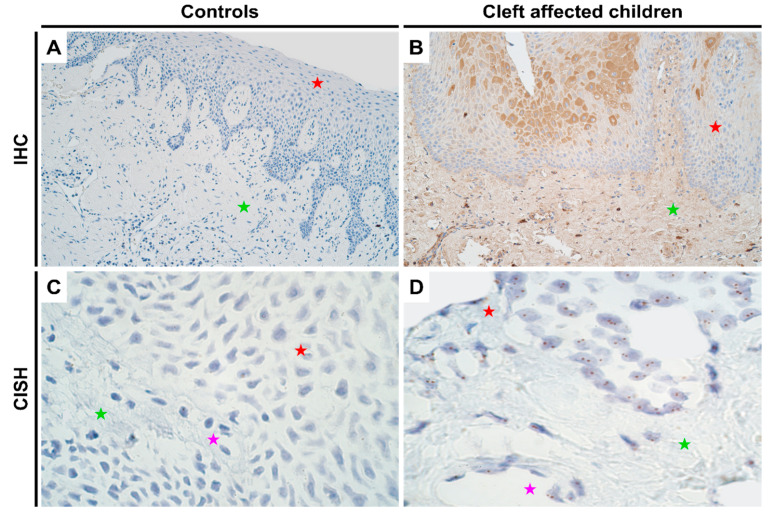
Expression of *HOXB3* gene visualized using IHC and CISH methods in controls and cleft patients. Protein expression of the *HOXB3* gene was visualized using immunohistochemistry (IHC) in (**A**) controls and (**B**) cleft affected children. (**A**) No positively stained (0) structures are visible in tissue obtained from controls; (**B**) Moderate number (++) of weakly stained epitheliocytes and connective tissue cells are seen in the cleft tissue obtained from a five-month-old girl. Original magnification, 200×. Gene amplification levels of *HOXB3* gene was visualized using chromogenic in-situ hybridization (CISH) in (**C**) controls and (**D**) cleft affected children. (**C**) No gene amplification (0) is seen in the tissue obtained from controls; (**D**) No gene amplification (0) is seen; however, gene expression is seen by brown dots in the nucleus of the epithelial, endothelial, and connective tissue cells in the cleft tissue obtained from a three-month-old girl. Original magnification, 1000×. Red star indicates epithelium; green star indicates connective tissue, while purple star indicates endothelial cells.

There were no notable differences in the genetic expression of *HOXB3* between normal and cleft affected mucosa. In both the tissues, all cell types (epithelial, endothelium and connective tissue cells) showed no gene amplification though gene expression was noted (Figure 2C,D; Supplementary Table S2). In terms of protein expression, like *DLX4*, controls showed no protein expression (Figure 2A; IHC). However, in the cleft tissue, there was a significant increase in protein expression in both epithelium (*p* < 0.001) and connective tissue cells (*p* < 0.001) with a mean IHC semi-quantitative grade of 2.1+ and 1.1+, respectively (Figure 2B and Table 1; Mann-Whitney U test). Further, epithelial cells showed a significantly higher expression of *HOXB3* when compared with connective tissue cells in the cleft tissue in terms of protein expression (*p* = 0.000; paired Wilcoxon signed rank test).

### 2.3. Gene and Protein Expression Analysis of MSX2 Gene in Control and Cleft Tissue

**Figure 3:**
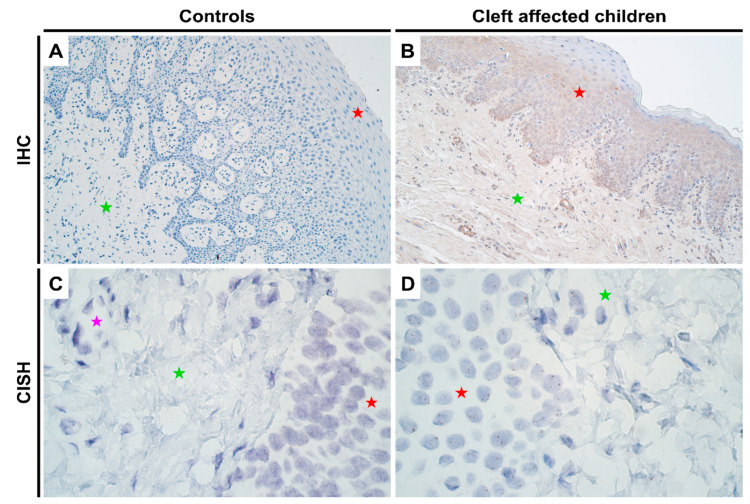
Expression of *MSX2* gene visualized using IHC and CISH methods in controls and cleft patients. Protein expression of the *MSX2* gene was visualized using immunohistochemistry (IHC) in (**A**) controls and (**B**) cleft affected children. (**A**) No positively stained (0) structures are visible in tissue obtained from controls; (**B**) Moderate number (++) of weakly stained epitheliocytes and endotheliocytes are seen in the cleft tissue obtained from a six-month-old girl. Original magnification, 200×. Gene amplification levels of *MSX2* gene was visualized using chromogenic in-situ hybridization (CISH) in (**C**) controls and (**D**) cleft affected children. (**C**) No gene amplification (0) is seen in the tissue obtained from controls; (**D**) No gene amplification (0) is seen; however, gene expression is seen by brown dots in the nucleus of the epithelial, endothelial, and connective tissue cells in the cleft tissue obtained from a five-month-old boy. Original magnification, 1000×. Red star indicates epithelium; green star indicates connective tissue, while purple star indicates endothelial cells.

As with *HOXB3*, no notable differences in genetic expression of *MSX2* between normal and cleft affected mucosa was noted in any of the cell types (Figure 3C,D; Supplementary Table S2). In terms of protein expression, like the other two homeobox genes, no expression was visualized in controls using IHC (Figure 3A). However, there was a significant increase in protein expression in both epithelium (*p* = 0.000) and connective tissue cells (*p* = 0.010) in cleft tissue with a mean IHC semi-quantitative grade of 1.4+ and 0.6+, respectively (Figure 3B and Table 1; Mann-Whitney U test). Further, there were significant differences noted in protein expression between the epithelium and connective tissue cells in cleft tissue (*p* = 0.003; paired Wilcoxon signed rank test).

### 2.4. Protein Expression Analysis of NF-κB in Control and Cleft Tissue

**Figure 4:**
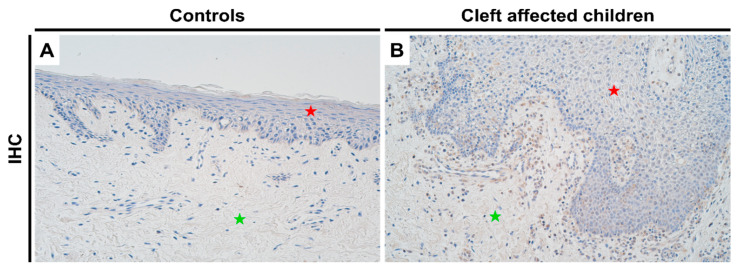
Expression of NF-κB protein complex visualized using IHC in controls and cleft patients. Protein expression of NF-κB was visualized using immunohistochemistry (IHC) in (**A**) controls and (**B**) cleft affected children. (**A**) No positively stained (0) structures are visible in tissue obtained from controls; (**B**) Moderate number (++) of positively stained inflammatory cells are seen in the cleft tissue obtained from a three-month-old boy. Original magnification, 200×. Red star indicates epithelium while green star indicates connective tissue.

For NF-κB, control tissues showed no protein expression (Figure 4A), while there was a significant increase in the expression in cleft tissue (Figure 4B) in both epithelial (*p* = 0.0001) and connective tissue cells (*p* = 0.003), with a mean IHC semi-quantitative grade of 1.7+ and 1.1+, respectively (Table 1; Mann-Whitney test). A paired Wilcoxon signed rank test revealed no significant differences between NF-κB expression in epithelial and connective tissue cells in the cleft tissue (*p* = 0.051).

### 2.5. Gene Expression Analysis of PTX3 Gene in Control and Cleft Tissue

**Figure 5:**
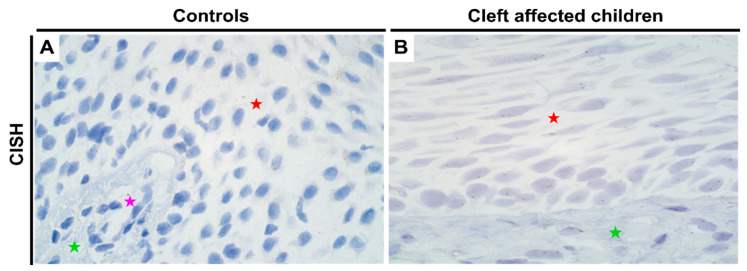
Expression of *PTX3* gene visualized using CISH in controls and cleft patients. Gene amplification levels of the *PTX3* gene was visualized using chromogenic in-situ hybridization (CISH) in (**A**) controls and (**B**) cleft affected children. (**A**) No gene amplification (0) is seen in the tissue obtained from controls; (**B**) No gene amplification (0) is seen; however, gene expression is seen by brown dots in the nucleus of the epitheliocytes and connective tissue cells in the cleft tissue obtained from a five-month-old girl. Original magnification, 1000×. Red star indicates epithelium; green star indicates connective tissue, while purple star indicates endothelial cells.

For *PTX3*, no changes in gene amplification or expression were noted between the control and cleft tissue (Table 1; Supplementary Table S2). All three cell types—epithelial, connective tissue, and endothelial cells showed equal expression of *PTX3* gene in both tissues (Figure 5A,B, respectively).

### 2.6. Correlation between Protein Expression of Homeobox Genes and NF-κB (IHC)

**Figure 6:**
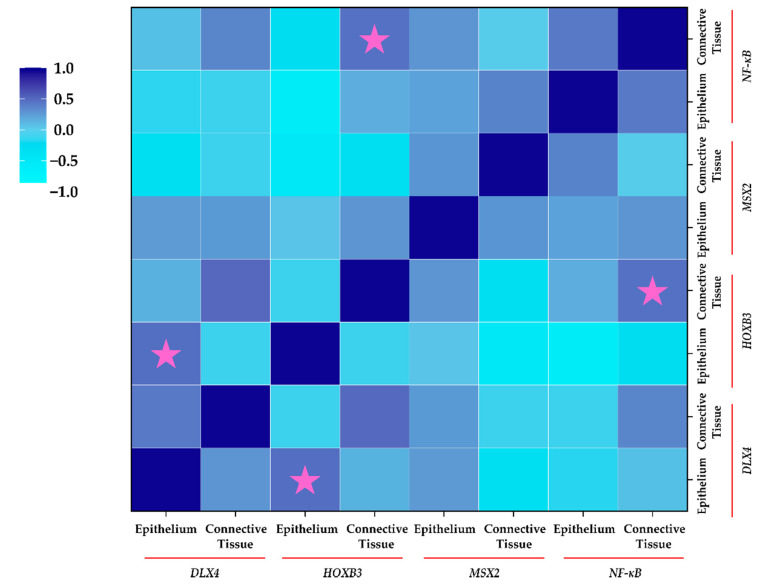
Correlation between protein expression of homeobox genes and NF-κB using IHC in cleft patients. Spearman’s Rho was used for correlation analysis. The legend for correlation is displayed on the left. Pink stars indicate significant correlation in expression (*p* < 0.05).

Correlation analysis (Figure 6) revealed a significant positive moderate correlation between epithelial expression of *DLX4* and *HOXB3* in cleft tissue (ρ = 0.472; *p* = 0.044). Similarly, a significant moderate positive correlation between connective tissue expression of *HOXB3* and NF-κB was noted (ρ = 0.455; *p* = 0.028). Interestingly, epithelial cells showed a non-significant strong negative correlation between *HOXB3* and NF-κB (ρ = −0.586; *p* = 0.102), indicating the differential expression and correlations between different types of cells in the cleft tissue.

## 3. Discussion

### 3.1. DLX4 Overexpression Leads to Increased Cellular Proliferation and Chronic Tissue Inflammation

Studies in mouse models have demonstrated that *DLX4* is expressed in the mesenchymal tissue of the maxilla just prior to the time of palatal closure [14]. The expression levels then undergo significant reduction at later stages of palatal closure, highlighting the crucial regulatory role played by the gene in palatogenesis [14]. Additionally, the *DLX* genes have been shown to be closely related to normal tooth development. In a study on dental pulp cells isolated from human wisdom teeth (which are still under development after birth), the authors reported the presence of a high expression of *DLX4* in crown completed and root completed stages of odontogenesis [41].

**Figure 7:**
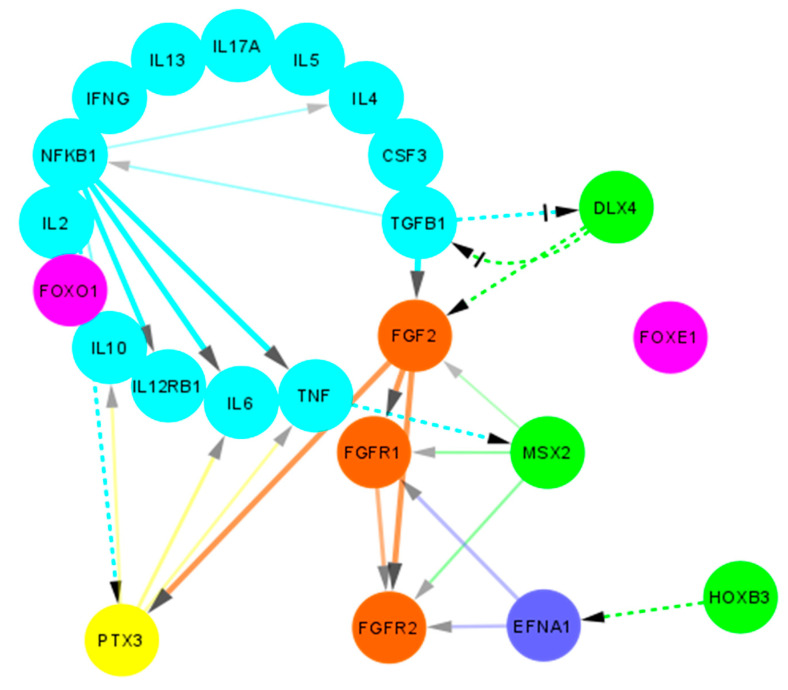
Protein-protein interactions between candidate genes and cytokines in non-syndromic cleft palate and lip. A non-comprehensive simplified overview of the protein-protein interactions as obtained using STRING (adapted and modified from https://www.string-db.org/, accessed on 28 September 2021) is shown. Light blue bubbles indicate cytokines, green bubbles indicate proteins encoded by homeobox genes, orange bubbles indicate FGF/FGFR pathway proteins, pink bubbles indicate FOX pathway proteins, dark blue bubble indicates Ephrin A1 while yellow bubble indicates the PTX3 protein. Solid lines indicate interactions obtained from STRING database. Dashed lines indicate potential interactions obtained from literature. Arrow heads indicate the direction of interaction. Crossed head indicate negative regulation.

Apart from the oral cavity, *DLX4* expression has been hugely discussed in the context of the human placenta and endometrium. The expression of the gene has been reported to be differentially elevated in the proliferative human endometrium, especially in the epithelium cells, which then reduces during the secretive phase [42]. This is quite interesting since a similar analogy can be attributed during palatogenesis, with preferential expression of *DLX4* in the epithelium cells (as reported in our results as well). Additionally, ectopic (or residual in case of clefting) expression of *DLX4* has been shown to increase cell proliferation by the inhibition of TGF-β/Smad signaling in cells, thereby causing p15 and p21 expression inhibition [9,43], which correlates with our previous findings whereby we found an increased detection of the proliferative marker Ki-67 [44], coupled with a pathological reduction in TGF-β1 expression in the cleft mucosa (Figure 7) [26].

While *DLX4* gene expression has been associated with stimulatory effects on cell mobility, migration, and invasion abilities in breast tumor and preeclampsia placenta [45,46], other lung cancer studies have shown the opposite [47], which makes it difficult to elucidate its role in cleft tissue. Future studies investigating this role may prove to be instrumental in understanding neural crest cell migration patterns and regulation. Furthermore, the gene has been shown to be in involved in epithelial-mesenchymal transition (EMT) whereby the epithelial cells lose their polarity and assume a mesenchymal phenotype [46,48]. Such transition is necessary for normal embryonic development, tissue remodeling, and wound repair. In terms of inflammation regulation, *DLX4* interacts directly with IL-1β that further leads to the downstream stimulation of *NF-κB* [49,50]. Moreover, IL-1β and *NF-κB* axis furthers stimulates multiple other targets including IL-6 and IL-8 (Figure 7) [51]. In the present study we also found an elevated *NF-κB* protein expression, which can be coupled with our previous results where we reported an elevated and consistent expression of IL-6 and elucidated its pro-inflammatory role in cleft tissue [26]. Additionally, stimulation of *NF-κB* by IL-1β and TNF-α causes suppression of TGF-β/Smad signaling which further exacerbates the pro-inflammatory environment [52].

Interestingly, interactions between *DLX4* and *bFGF/FGF2* have been reported in ovarian cancer tissue. *DLX4* has been shown to directly stimulate expression of both the low and high molecular weight isoforms of *FGF2* (Figure 7) which in turn stimulate VEGF (vascular endothelial growth factor) and promote an angiogenetic environment [53]. We also reported an increase in *FGF2* expression in cleft tissue [40] which can lead to accelerated wound healing (*FGF2* can further promote EMT and wound closure). Another possible pathway for angiogenetic promotion has been postulated by the means of *DLX4* induced expression of iNOS (inducible nitric oxide synthetase) [54]. This promotion of angiogenetic and pro-inflammatory environment by *DLX4* gives it a rather unusual protective role in the cleft tissue.

Finally, a regulatory mechanism between TGF-β and *DLX4* has been reported. Induction of TGF-β1 has been shown to reduce *DLX4* levels in human dental pulp cells, which leads to impaired iPSC (induced pluripotent stem cells) generation [41]. These findings are quite interesting, since we suspect the loss (or significant reduction) of TGF-β expression (which could be due to non-attenuation of *DLX4* expression at the time of palatal closure) leads to sustained residual expression of *DLX4,* which ironically suppresses TGF-β signaling even more, thereby creating an auto-sustained loop (Figure 7).

### 3.2. Overexpression of HOXB3 Co-Stimulates Cellular Proliferation and Angiogenesis

The *HOXB3* gene has been implicated in the promotion of angiogenesis via upregulating the expression of Ephrin A1 (*EFNA1*; Figure 7) and is needed to induce capillary morphogenesis in the endothelial sprouts [55]. Interactions between the *EFNA1* and *FGF/FGFR* pathways have been shown by many authors in the past [56,57,58], which would make it an interesting link to investigate in the context of the cleft lip and palate. The expression of *HOXB3* has also been implicated in the hematopoietic pathways, wherein its expression is significantly higher in primitive CD34+ cells, which eventually gets downregulated as the cells differentiate into committed progenitors [59]. In a study in knockout mice, the authors demonstrated that the *HOXB3* gene plays a direct physiological role in regulating stem cell regeneration in which its expression was critical to the achieving of maximum proliferation rates of these progenitor cells [60].

This role of *HOXB3* in controlling cell proliferation has also been established in the context of cleft lip and palate. In a study on regulatory network analysis of cleft genes, Li et al., reported that *HOXB3* was enriched in the process of positive regulation of cell division, which is an important process in lip development [61]. Furthermore, the authors found that the downstream targets of the gene were involved in the wound healing processes and in the positive regulation of transcription from RNA polymerase II promoter, processes which are critical for palatogenesis [61].

Although in our study we found no significant differences in the genetic expression of the gene, protein expression analysis using IHC did show us a significant increase in the expression levels in cleft tissue. A critical moment in lip formation occurs just before the completion of the upper lips whereby the lateral nasal processes show a heightened session of cell division [62,63]. Disturbances in this process can result in clefting. Since both *HOXB3* and *DLX4* appear to accelerate cell proliferation, it is interesting to find a moderate positive correlation between the expression of the two genes in the cleft epithelial tissue, indicating some underlying signaling between the two genes which requires further investigation.

### 3.3. MSX2 Overexpression Causes Disturbances in Normal Tooth and Bone Development

In odontogenesis (formation of teeth), amelogenesis is a key process through which the enamel is formed. For successful amelogenesis to occur, dentinogenesis (formation of dentin) must occur. A precursor event to both these processes is the interaction between the epithelial and mesenchymal cells that allows the former to differentiate into ameloblasts which produce the enamel while the later differentiates into odontoblasts which produce the dentin [64,65]. Multiple case reports have been published showcasing the high prevalence of tooth anomalies in cleft affected children [66,67,68]. In cleft children predominantly enamel defects have been reported in the maxillary permanent central incisors which are adjacent to the clefts, with the intensity of defects correlating strongly with the severity of the clefting [66,67,68]. Studies in mice and humans have shown that silencing of the *MSX2* gene due to mutations leads to amelogenesis imperfecta [69,70,71]. Furthermore, overexpression of *MSX2* also leads to dental anomalies [72], which highlights the crucial role of the gene in cleft patients.

Apart from its role in tooth development, *MSX2* also plays a crucial role in bone formation. Overexpression of *MSX2* (as seen in our study using IHC) caused by sustained TNF-α levels (shown in previous study; Figure 7) [26] leads to *NF-κB* activation which in turn inhibits the bone morphogenetic protein 2 (BMP2)-induced expression of alkaline phosphatase (ALP) enzyme [73], thereby hindering bone formation. Interestingly, in another study we found a statistically significant decrease in *BMP2* and *BMP4* expression in bilateral cleft lip and palate tissue [74], which highlights the role of *MSX2* in alveolar bone regeneration and the remodeling process. Additionally, interactions between *MSX2* and *FGF2* in bone formation abnormalities have also been reported [75,76]. In a study in mouse mammary epithelial cells, Bari et al., demonstrated that *MSX2* expression downregulated epithelial marker E-cadherin expression and caused an upregulation of the mesenchymal markers vimentin and N-cadherin [77]. Furthermore, the authors showed that *MSX2* promoted EMT in epithelial cells [77], which is essential during midline fusion of the palatal shelves.

### 3.4. Overexpression of NF-κB Promotes Chronic Inflammation and Bone Resorption

Apart from the known role of *NF-κB* in inflammation promotion, it has also been implicated in other processes seen in cleft patients. Bone formation and maintenance is a dynamic process that is under the regulatory control of various factors. While we noted previously an increase in *FGF/FGFR* pathways [40] which promote osteoblast differentiation, a chronic inflammatory state coupled with decrease in BMPs and TGF-β1/3 expression [26,74] usually results in greater bone resorption in cleft patients. This process is further compounded by an increase in the *NF-κB* expression due to activation of the classical pathway by TNF-α, IL-1β, IL-6, and IL-17 produced by T cells and other cells, all of which have been reported to be elevated in cleft tissue [26].

Like *MSX2*, *NF-κB* overexpression has also been associated with dental anomalies. In a study using the mice model, Blackburn et al., showed that *NF-κB* overexpression induces an ectopic odontogenesis program that is usually suppressed under physiological conditions [78]. Furthermore, they found that mice overexpressing *NF-κB* show supernumerary teeth [78], a complication which was found to be the third most common dental anomaly affecting as much as 16% of cleft affected children [79].

### 3.5. PTX3 Overexpression can Lead to Dysregulated Wound Healing

The pentraxins are a large highly conserved superfamily of pattern recognition proteins that are major components of the humoral arm of the innate immune system [80]. The commonly known pentraxins include the C-reactive protein (CRP) and serum amyloid P component (SAP), both of which unlike *PTX3*, are short pentraxins produced in the liver [80]. *PTX3* has been shown to be upregulated in response to a rise in transcriptional factors such as *NF-κB* in monocytes, macrophages, and endothelial cells (Figure 7) [80]. Furthermore, it has been shown to attenuate excessive macrophage-mediated inflammatory activity, thereby promoting the wound healing processes [80]. Additionally, overexpression of *PTX3* gene has been demonstrated in mice to be associated with severe tissue injury and lethality, especially after ischemic events [81]. This enhanced lethality has been attributed to the exacerbated inflammatory response and the production of *PTX3* mediated pro-inflammatory cytokines, especially TNF-α [81]. In the present study, although no change in gene expression was noted, there may be differences in protein expression that need further evaluation.

Apart from regulating the inflammatory responses, *PTX3* has also been shown to play a crucial role in tissue remodeling following an injury. In mice models it has been demonstrated that deficiency of *PTX3* leads to altered thrombotic responses coupled with increased deposition of both fibrin and collagen [82,83,84]. Moreover, *PTX3*-deficient macrophages and fibroblasts were associated with defective fibrinolytic activity [82]. The deficiency of the gene has been further shown to suppress osteoblast function leading to a lower bone volume in mice models [85]. Due to its *FGF2* binding properties, it acts as a bone protective factor which is important for osteoblast maturation by antagonizing the negative effects of *FGF2* during early stages of bone formation [85]. *PTX3* expression has also been linked to promote EMT in human proximal tubular epithelial cells, thereby inducing fibrotic changes in the renal tissue [86].

### 3.6. Limitations of the Present Study

The relevance and importance of the studies like the present one has been discussed and reported previously [40]. However, the results obtained in the present study are constrained by certain limitations. First, the limited number of control and cleft patients in the present study limits our ability to generalize our findings. This is rather understandable due to genuine concerns of the parents, given the tender age at which corrective surgery is undertaken [26,40]. Secondly, the complex etio-pathogenesis of cleft lip and palate makes it difficult to predict or state whether neonatal gene expression resembles embryonic gene expression, especially during the events just prior and during the palatal closure. Thirdly, there is extremely limited data in terms of comparison with the two stages of dentition in humans—the primary or milk dentition and the mixed dentition stages, which makes it difficult to analyze the gene expression and interactions. Fourth, gene-gene interactions (including gene products and transcriptional factors) are difficult to elucidate completely, even more so in the setting of a polyfactorial etiology. Finally, the role of environmental factors like maternal and paternal family history, lifestyle etc. have profound effects on the manifestations and clinical course of the condition, both of which are difficult to ascertain. Nonetheless, the results obtained in the present study can serve as a baseline for future studies to explore the molecular and phenotypic connections between various cytokines, candidate genes, and tissue repair genes.

## 4. Materials and Methods

### 4.1. Demographic Profile of the Study Participants

In the present study, lip mucosal tissue samples were obtained from 15 pediatric children (seven female children and eight male children) who reported at the Department of Oral and Maxillofacial Surgery, Institute of Stomatology, Riga Stradiņš University (RSU), Latvia, for consultation and treatment of unilateral non-syndromic congenital cleft lip and/or palate (Table 2). The tissue material was collected during the corrective plastic surgery from the site of clefting by the same surgeon. None of the children were previously diagnosed with coexisting genetic syndromes, chromosomal abnormalities, or immune deficiencies. Additionally, seven (three female children and four male children) control pediatric patients were enrolled. These children reported to the department for correction of low attached upper lip frenulum. The lip mucosal tissue material was obtained during upper labial frenectomy and was free of any pathology (including clefting) and/or inflammation. The control children were aged between five and six years.

### 4.2. Ethical Permission and Consent for Participation

The study design and protocol were approved by the Research Ethics Committee (REC) of RSU wide approval no. 5/28.06.2018, per the guidelines in the 1975 Declaration of Helsinki (revised in 2008). The study protocol additionally complied with the frameworks of the local and EU ethical norms. Written and oral consents from all pediatric patients were obtained from the parents/legal guardians for participation in the study. Simultaneous consent for publication of the study data was also obtained.

### 4.3. Sample Collection and Processing

Tissue samples collected during the corrective surgery (plastic surgery for cleft affected children and frenectomy for control children) were fixed for a day in a mixture of 2% formaldehyde and 0.2% picric acid in 0.1M phosphate buffer (pH 7.2). Prior to paraffin embedment, the samples were treated and rinsed with 10% saccharose containing Tyrode buffer for 12 h. The samples were then registered and assigned randomized internal codes. Only the internal codes and patient history (as shown in Table 2) was kept and disclosed to the researchers and/or lab assistants.

### 4.4. Slide Preparation and Visualization of Routine Histological Staining

Serial tissue sections of 3–4 µm were prepared from the solidified paraffin block for routine hematoxylin and eosin staining (H&E) and immunohistochemistry (IHC). Tissue sections were loaded on the slides and kept for 20–60 min in a thermostat at 56 °C. De-paraffinization of the sections was done using 96% ethanol and xylene solutions before H&E staining (Mayer’s; Bio Optica Milano, Milan, Italy). Slides were dehydrated with ethanol and clarified with carboxylol and xylene. A drop of histological Pertex glue (Histolab Products AB, Askim, Sweden) was applied and slides were covered with a cover glass. Visualization of the slides was done using Leica DC 300F brightfield light microscope (Leica DM500RB; Leica Biosystems Richmond, Richmond, IL, USA).

### 4.5. Biotin-Streptavidin Immunohistochemistry (IHC)

Prepared tissue sections (de-paraffinized, washed, and cleared) were rinsed for 10 min with TRIS wash buffer (Diapath, Martinengo, Italy) and then boiled in EDTA buffer for 10 min. The tissue sections were then cooled to 65 °C before second placement in TRIS wash buffer. 3% peroxidase (Dako, Naestved, Denmark) was used to block the activity of the endogenous peroxidase. Antibody diluent (Cell Marque^TM^, Rocklin, CA, USA) was used to dilute the antibodies. The tissue sections were incubated with primary antibodies for 2 h in accordance with manufacturer’s protocols (Table 3). The sections were then washed with TRIS wash buffer. The HiDef DetectionTM HRP polymer system (Cell Marque^TM^, Rocklin, CA, USA) was used for rabbit antibodies as per the manufacturer’s guidelines.

Tissue sections were subsequently incubated with biotin-containing secondary antibody for 30 min followed by rinsing for 10 min in TRIS wash buffer. This step was repeated for incubation with tertiary antibody. DAB+ chromogenic liquid was used to coat the tissue sections (DAB Substrate Kit, Cell Marque^TM^, Rocklin, CA, USA). Finally, the sections were incubated at room temperature for 10 min to obtain brown staining of immunoreactive structures. The sections then underwent washing using distilled water and were counter-stained with haematoxylin for 2 min. Dehydration was performed using ethanol solution followed by clarification using carboxylol and xylene. The slides were then visualized using a light microscope (described in previous section). Negative and positive IHC controls were also prepared for each sample in the study.

### 4.6. Chromogenic In-Situ Hybridization (CISH)

Pretreatment and denaturation of the sections was done following standard laboratory protocol using the ZytoDot 2C CISH Implementation Kit (ZytoVision GmbH, Bremerhaven, Germany). CISH probes of *DLX4, HOXB3, MSX2*, and *PTX3* genes were used in the present study (Empire Genomics, Williamsville, NY, USA). Hybridization was done using 10 µL of each probe followed by covering of the slides using coverslip. The slides were then placed on a hot plate for 5 min at 79 °C, and transferred to a humidity chamber overnight at 37 °C. On the following day, the coverslips were removed, and DAB solution was applied to the slide per the manufacturer’s protocol. The nucleus was counterstained with a nuclear dye and dehydration using increasing gradients of ethanol was done. Cover slip was placed, and the hybridized probe fragments were visualized using brightfield microscope. Under the microscope, two brown coloured dots (signals) were expected per nuclei of normal cells in interphase or metaphase without aberrations of the examined chromosomes. Due to limited availability of tissue material, only three control samples were used for CISH (1 female child and 2 male children).

### 4.7. Semi-Quantitative Grading and Statistical Analysis

The slides were analyzed under the microscope and with Image Pro Plus 6.0 (Media Cybernetics, Rockville, MD, USA). For IHC reactions, cells with brown nucleus/cytoplasm were considered as immunoreactive or immunopositive. Semi-quantitative grading of the epitheliocytes and connective tissue cells was done by two independent morphologists in at least five randomly selected vision fields at 400× magnification. For CISH, brown signals were evaluated per nuclei in at least 30 cells of each type (epitheliocytes, endotheliocyte, and connective tissue cells) at 1000× magnification using immersion oil. A grading scale interpretation table developed in our previous study was used for evaluating IHC and CISH results (Supplementary Tables S1 and S2) [40].

Statistical analysis was performed using non-parametric related samples Wilcoxon signed rank test for comparison within different cells (intra-group analysis). For control vs. cleft tissue comparison, a Mann Whitney U test and one-sample Wilcoxon signed rank test were used (inter-group analysis). Spearman’s rho was used for correlation analysis. Statistics were done using SPSS v26.0 (IBM Corp., Armonk, NY, USA). For statistical analysis, the numbers of “+” values were considered as equivalent to absolute whole numbers (e.g., “+” corresponded to 1; “++” corresponded to 2, and so on). Statistical significance was set at *p* < 0.05.

## 5. Conclusions

The following three conclusions are noteworthy from our present study:

1. Residual expression of *DLX4* upregulates the expression of *NF-κB* in cleft mucosa leading to increased cellular proliferation and promotion of a pro-inflammatory environment. This accelerated proliferative state is further stimulated by the elevated *HOXB3* expression.
2. Elevated expression of *MSX2* and *NF-κB* in the cleft-affected lip tissue seems to negatively affect critical developmental pathways, resulting in the formation and persistence of a dysregulated hard tissue postnatally, a finding commonly reported in the cleft patients.
3. The *PTX3* gene plays a crucial role in regulating and fine-tuning the persistent inflammatory responses characteristically seen in the postnatal cleft affected lip mucosa.

## Figures and Tables

**Table 1:** Average semi-quantitative score of the results obtained from IHC and CISH.

Groups	Immunohistochemistry (IHC)				Chromogenic In-Situ Hybridization (CISH)			
	*DLX4*	*HOXB3*	*MSX2*	*NF-κB*	*DLX4*	*HOXB3*	*MSX2*	*PTX3*
Controls								
Epithelium	0	0	0	0	ND *	0	0	0
Connective Tissue	0	0	0	0	ND *	0	0	0
Endothelium	NT	NT	NT	NT	ND *	0	0	0
Cleft affected children								
Epithelium	1.7+	2.1+	1.4+	1.7+	0	0	0	0
Connective Tissue	1.6+	1.1+	0.6+	1.1+	0	0	0	0
Endothelium	NT *	NT *	NT*	NT *	0	0	0	0

* NT stands for not tested while ND stands for not determined (refer to Supplementary Tables S1 and S2 for interpretation).

**Table 2:** Clinical diagnosis and demographic profile of the cleft affected children.

Patient Number	Age (Months)	Gender	Clinical Diagnosis ^†^	Anamnesis
1	3	F_c_	Cheilognathouranoschisis sinistra	-
2	3	M_c_	Cheilognathouranoschisis dextra	-
3	3.5	M_c_	Cheilognathouranoschisis sinistra	Mother reported use of paracetamol during pregnancy; father was smoker and partially alcoholic. Epilepsy in the family tree. Child was born overweight
4	4	M_c_	Cheilognathouranoschisis sinistra	There was a reported threat of miscarriage in the 36th gestational week; history of clefts in the family tree.
5	4	F_c_	Cheilognathouranoschisis dextra	-
6	4	F_c_	Cheilognathouranoschisis sinistra	Born in the 42nd gestational week; mother reported use of paracetamol during pregnancy
7	4	M_c_	Cheilognathouranoschisis sinistra	Born in the 41st gestational week; mother reported use of paracetamol during pregnancy
8	4	F_c_	Cheilognathouranoschisis sinistra	-
9	4	M_c_	Cheilognathouranoschisis sinistra	-
10	4	F_c_	Cheilognathouranoschisis sinistra	-
11	4.5	M_c_	Cheilognathouranoschisis sinistra	History of Down syndrome in the family tree
12	5	M_c_	Cheilognathouranoschisis sinistra	History of clefts in the family tree; mother reported use of Amoxiclav during pregnancy
13	5	F_c_	Cheilognathouranoschisis sinistra	Mother developed gestational diabetes during the pregnancy
14	6	F_c_	Cheilognathouranoschisis sinistra	-
15	8	M_c_	Cheilognathouranoschisis sinistra	Both parents were regular smokers

^†^ Clinical diagnosis is provided in Latin; cheilognathouranoschisis—cleft lip, alveolar ridge, and palate; sinistra—left; dextra—right. F_c_—female child; M_c_—male child.

**Table 3:** Description and characteristics of the primary antibodies.

Primary Antibody *	Antibody Characteristics **	Clone	Dilution	Catalogue No.	Manufacturer
*DLX4*	Polyclonal rabbit AB against human AG	-	1:100	orb626417	Biorbyt Limited (UK)
*HOXB3*	Polyclonal rabbit AB against human AG	H-50	1:100	sc-28606	Santa Cruz (USA)
*MSX2*	Polyclonal rabbit AB against human AG	-	1:100	ab223692	Abcam (UK)
*NF-κB* (p50/p105)	Monoclonal rabbit AB against human AG	E381	1:100	ab32360	Abcam (UK)

* Abbreviations: *DLX4*—Distal-Less homeobox 4; *HOXB3*—homeobox B3; *MSX2*—Msh homeobox 2 and *NF-κB*—nuclear factor kappa-light-chain-enhancer of activated B cells. ** Abbreviations: AB, antibody; AG, antigen.

## Data Availability

All results obtained from IHC and CISH are presented in the Supplementary Tables S1 and S2.

## Supplementary Materials

The following are available online at https://www.mdpi.com/article/10.3390/jpm11111135/s1, Table S1: Summarized dataset of results obtained from immunohistochemistry (IHC), Table S2: Summarized dataset of results obtained from chromogenic in-situ hybridization (CISH), Figure S1: Positive and negative controls for DLX4 IHC antibody, Figure S2: Positive and negative controls for HOXB3 IHC antibody, Figure S3: Positive and negative controls for MSX2 IHC antibody, Figure S4: Positive and negative controls for NF-κB IHC antibody.
