# Supplementary material for: Evaluating the Expression of Candidate Homeobox Genes and Their Role in Local-Site Inflammation in Mucosal Tissue Obtained from Children with Non-Syndromic Cleft Lip and Palate
Source: J Pers Med. 2021 Nov 2;11(11):1135. doi: 10.3390/jpm11111135 (PMC8618679; doi:10.3390/jpm11111135)
Supplement: Supplementary file 1 [file jpm-11-01135-s001.zip › jpm-1425000-supplementary.pdf]

Supplementary Table S1. Summarized dataset of results obtained from immunohistochemistry (IHC).

Patient Number	DLX4		HOXB3		MSX2		NF-κB	
	Epi	CT	Epi	CT	Epi	CT	Epi	CT
Cleft affected children								
1	++	++	++	+	+	0	+	0
2	++	+++	++	++	++	0	++	+++
3	+	++	++	+	+	+	++	+
4	+	0	++	+	++	+	++	+
5	+	0	++	+	0	0	++	+
6	+	++	++	+	+	+	0	+
7	++	+	+++	+	+	0	+	0
8	++	+	++	+	+	+	++	++
9	++	++	++	+	+	0	++	++
10	+++	++	+++	+	++	0	+	+
11	++	+	++	+	++	+	++	0
12	++	++	++	+	+	+	++	0
13	++	++	++	+	++	+	++	++
14	++	++	++	+	++	+	++	++
15	+	++	++	+	++	+	++	+
Control children								
1	0	0	0	0	0	0	0	0
2	0	0	0	0	0	0	0	0
3	0	0	0	0	0	0	0	0
4	0	0	0	0	0	0	0	0
5	0	0	0	0	0	0	0	0
6	0	0	0	0	0	0	0	0
7	0	0	0	0	0	0	0	0

† Abbreviations: DLX4 - Distal-Less Homeobox 4; HOXB3 - Homeobox B3; MSX2 - Msh Homeobox 2 and NF-κB - nuclear factor kappa-light-chain-enhancer of activated B cells. Epi – epitheliocytes; CT – connective tissue cells.

For interpretation of the semi-quantitative scale used for IHC, please refer below [Pilmane et al., 1998]:

0 No cells with a positive reaction were detected in the visual field

\+ Few cells with a positive reaction were detected in the visual field

++ Moderate number of cells with a positive reaction were detected in the visual field

+++ Numerous cells with a positive reaction were detected in the visual field

++++ Abundant cells with a positive reaction were detected in the visual field

Reference: Pilmane, M.; Rumba, I.; Sundler, F.; Luts, A. Patterns of distribution and occurrence of neuroendocrine elements in lungs of humans with chronic lung disease. Proc. Latv. Acad. Sci. **1998**, 52, 144–152.

Positive and Negative Controls for IHC

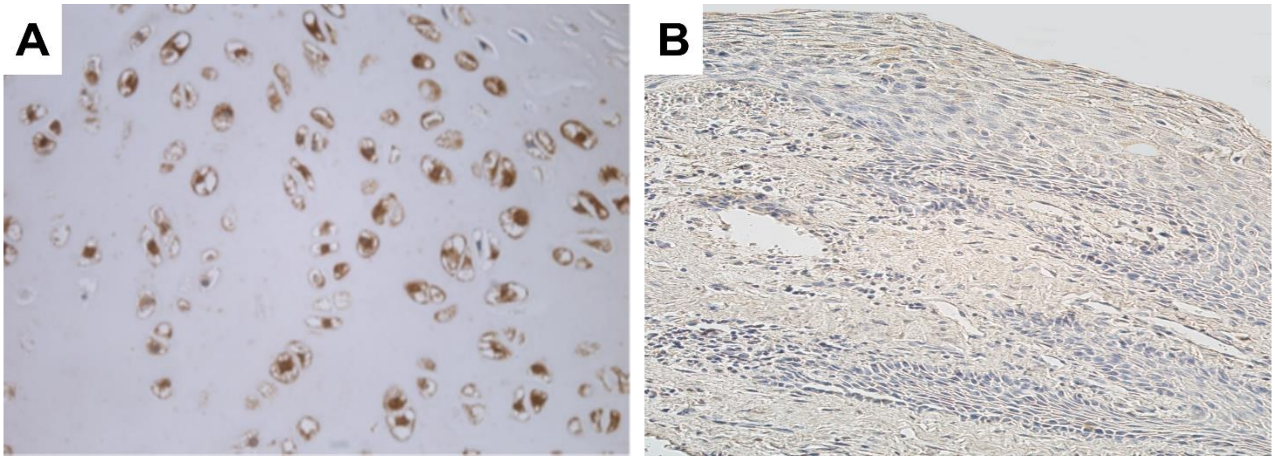

Supplementary Figure S1. Positive and negative controls for *DLX4* IHC antibody. (**A**) In the positive control note the presence of numerous *DLX4* positive chondrocytes in the hyaline cartilage of large bronchi in a patient with COPD (chronic obstructive pulmonary disease). Original magnification, 200×. (**B**) In the negative (water) control no positive cell structures are seen in the lip mucosal tissue. Original magnification, 250×.

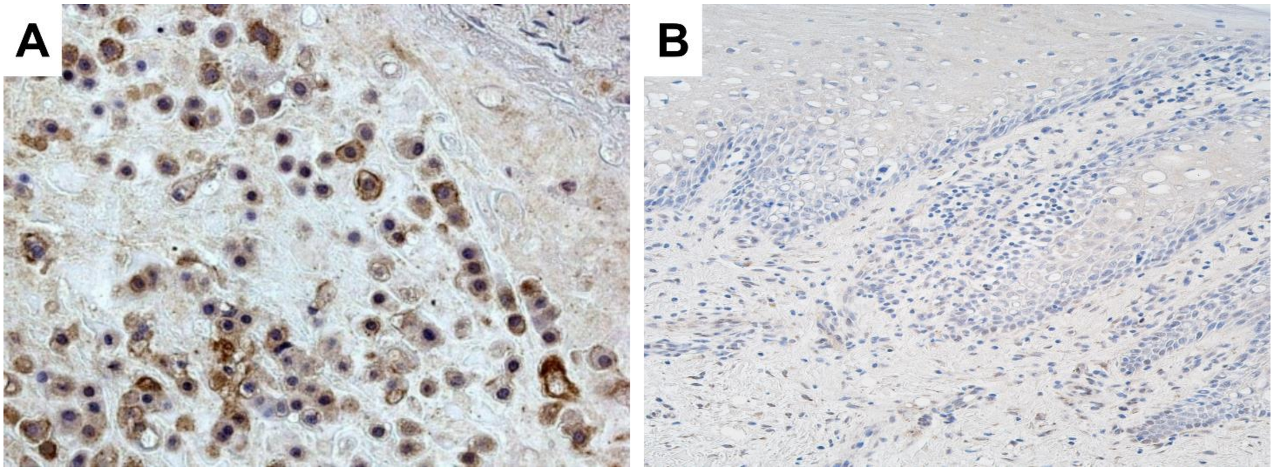

Supplementary Figure S2. Positive and negative controls for *HOXB3* IHC antibody. (**A**) In the positive control note the presence of numerous *HOXB3* positive Hoffbauer cells in the human placental tissue. Original magnification, 250×. (**B**) In the negative (water) control no positive cell structures are seen in the lip mucosal tissue. Original magnification, 200×.

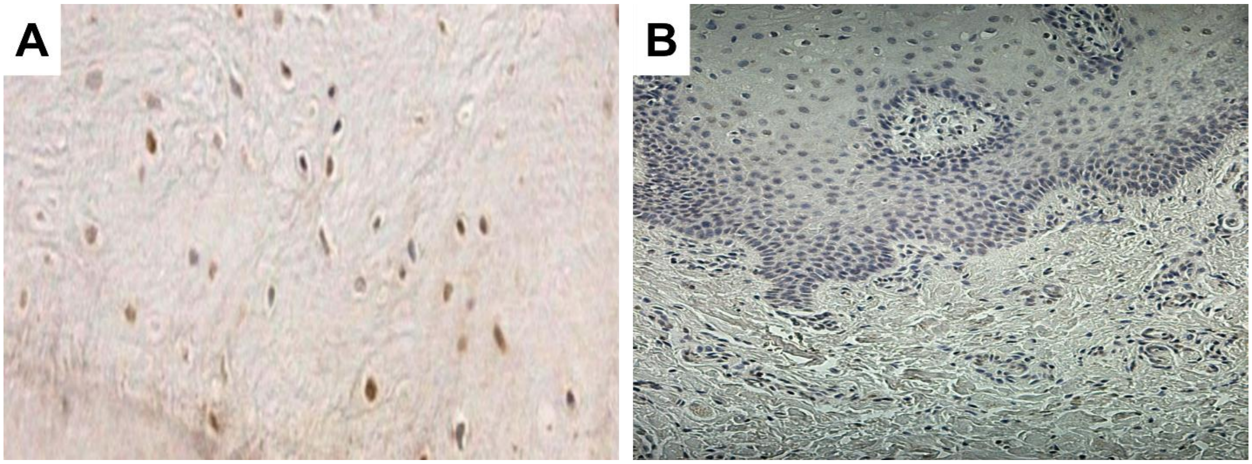

Supplementary Figure S3. Positive and negative controls for *MSX2* IHC antibody. (**A**) In the positive control note the presence of numerous *MSX2* positive osteocytes in the bone tissue from a 8-years old patient with temporomandibular joint ankylosis. Original magnification, 250×. (**B**) In the negative (water) control no positive cell structures are seen in the lip mucosal tissue. Original magnification, 200×.

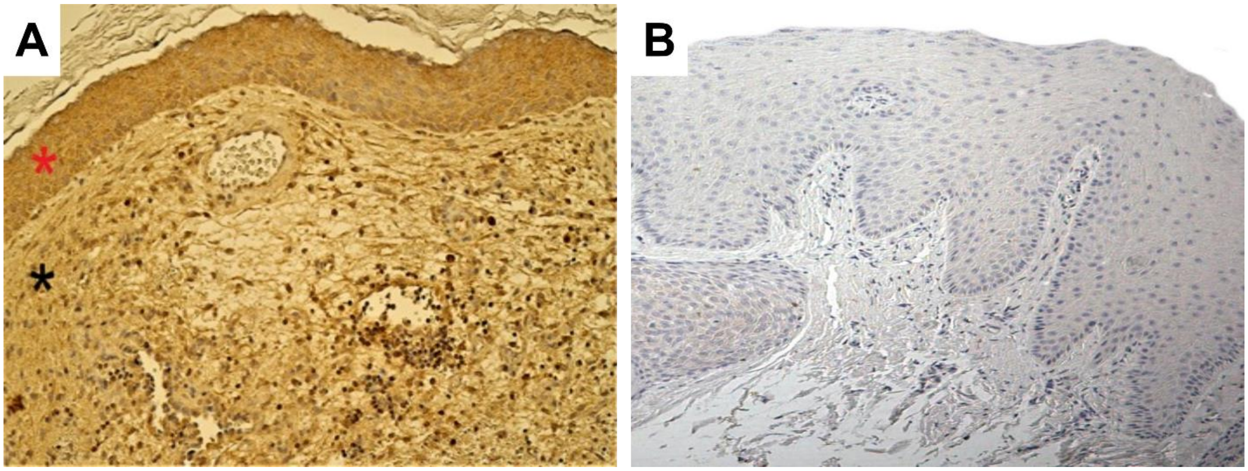

Supplementary Figure S4. Positive and negative controls for *NF-κB* IHC antibody. (**A**) In the positive control note the presence of moderate *NF-κB* positive cells in the epithelium (red star) and a few to moderate NF-κB positive cells in the connective tissue (black star) in the skin tissue obtained from a cholesteatoma patient. Original magnification, 200×. (**B**) In the negative (water) control no positive cell structures are seen in the lip mucosal tissue. Original magnification, 200×.

Supplementary Table S2. Summarized dataset of results obtained from chromogenic in-situ hybridization (CISH).

Patient Number	DLX4			HOXB3			MSX2			PTX3		
	Epi	CT	Endo	Epi	CT	Endo	Epi	CT	Endo	Epi	CT	Endo
Cleft affected children												
1	0	0	0	0	0	0	0	0	0	0	0	0
2	0	0	0	0	0	0	0	0	0	0	0	0
3	0	0	0	0	0	0	0	0	0	0	0	0
4	0	0	0	0	0	0	0	0	0	0	0	0
5	0	0	0	0	0	0	0	0	0	0	0	0
6	0	0	0	0	0	0	0	0	0	0	0	0
7	0	0	0	0	0	0	0	0	0	0	0	0
8	0	0	0	0	0	0	0	0	0	0	0	0
9	0	0	0	0	0	0	0	0	0	0	0	0
10	0	0	0	0	0	0	0	0	0	0	0	0
11	0	0	0	0	0	0	0	0	0	0	0	0
12	0	0	0	0	0	0	0	0	0	0	0	0
13	0	0	0	0	0	0	0	0	0	0	0	0
14	0	0	0	0	0	0	0	0	0	0	0	0
15	0	0	0	0	0	0	0	0	0	0	0	0
Control children												
1	-	-	-	0	0	0	0	0	0	0	0	0
2	-	-	-	0	0	0	0	0	0	0	0	0
3	-	-	-	0	0	0	0	0	0	0	0	0

† Abbreviations: DLX4 - Distal-Less Homeobox 4; HOXB3 - Homeobox B3; MSX2 - Msh Homeobox 2 and NF-κB - nuclear factor kappa-light-chain-enhancer of activated B cells. Epi – epitheliocytes; CT – connective tissue cells.

For interpretation of the semi-quantitative scale used for CISH, please refer below [Pilmane et al., 2021]:

Assigned Value	In-Lab Criteria Used for Assignment of Value	Interpretation
0	1 to 5 brown signals (copies) per nucleus detected in the cells	No amplification
+	5 to 6 brown signals (copies) per nucleus detected in the cells	Low-level amplification
++	6 to 10 brown signals (copies) per nucleus detected in the cells	Moderate-level amplification
+++	>10 brown signals (copies) detected in the cells	High-level amplification
++++	Large brown of green signals (copies) per nucleus detected in the cells	High-level amplification

**** Please note:** Fields in CISH Table S2 showing "-" indicates no detection of signals i.e., no brown signals were seen in the nucleus of the cells. Such sections were labelled as "negative and non-amplified" indicating no gene expression.

Reference: Pilmane, M.; Jain, N.; Vitenberga-Verza, Z. Expression Analysis of *FGF/FGFR* and *FOX* Family Proteins in Mucosal Tissue Obtained from Orofacial Cleft-Affected Children. *Biology* **2021**, *10*, 423. https://doi.org/10.3390/biology10050423.
